# Neurological Complications following Surgical Treatments of the Lower Molars

**DOI:** 10.1155/2024/5415597

**Published:** 2024-09-09

**Authors:** Antonio Mancini, Angelo Michele Inchingolo, Marco Di Blasio, Elisabetta de Ruvo, Angela Di Noia, Laura Ferrante, Gaetano Del Vecchio, Andrea Palermo, Francesco Inchingolo, Alessio Danilo Inchingolo, Gianna Dipalma

**Affiliations:** ^1^ Department of Interdisciplinary Medicine School of Medicine University of Bari “Aldo Moro”, Bari 70124, Italy; ^2^ Department of Biomedical Surgical and Dental Sciences University of Milan, Milan, Italy; ^3^ College of Medicine and Dentistry, Birmingham, UK

## Abstract

**Aim:**

The current review aims to explore postoperative neurological complications in third molar extractive surgery.

**Materials and Methods:**

The PRISMA protocols were followed when conducting this review. We found a total of 2,250 articles that matched our topic using the Boolean keywords, mandibular nerve complications AND oral surgery, from PubMed (1,083), Scopus (435), and Web of Science (732), with the filters of English language articles, time range January 1, 2003, to September 30, 2023, and human studies. After 762 duplicates were eliminated, there remained 1,488 articles. Eleven final articles were deemed of the highest relevance to our topic by eliminating articles in animals, non-English language, reviews, meta-analysis, and off-topic. A potential risk in the third molar extraction was temporary loss of sensibility often caused by mild compression or irritation of the mandibular nerve. This typically resolves within weeks or months, but in severe cases, recovery might take longer. Permanent loss of sensation can occur, indicating significant nerve damage and lasting effects on touch, temperature, or pain perception.

**Conclusions:**

Various treatments exist for nerve damage, including low-level laser therapy, pain management medications, or physical therapy. While these therapies may improve neurosensory impairment, patients often report a decline in their quality of life.

## 1. Introduction

Lower molar disinclusion surgery is a common dental procedure that involves the extraction of third molars that are partially or completely included in the mandibular bone. Although generally considered safe, this operation can involve a number of neurological complications, which vary in severity and can significantly affect the patient's quality of life.

Postoperative neurological complications are a major concern for oral and maxillofacial surgeons.

Major complications include damage to the inferior alveolar nerve (IAN) and lingual nerve (LN) [1]. The IAN, which runs along the mandible, is particularly susceptible to injury during removal of the lower included molars [2, 3, 4]. Damage to this nerve can manifest with symptoms such as paresthesia, anesthesia, dysesthesia, and, in rare cases, neuropathic pain [3, 5, 6, 7, 8].

The main branches of the LN provide sensitivity to various areas, including the jaw [9]. The IAN is responsible for innervating the lower teeth, gums, oral mucosa, and part of the skin of the jaw itself [10, 11]. This branch transmits tactile, thermal, and pain sensitivity signals from the jaw [12]. Thanks to the LN, innervate the front part of the tongue and part of the floor of the mouth. Contributing to the sensitivity of the tongue and the associated oral mucosa [13], thanks to the buccal nerve, it provides sensitivity to the cheek mucosa and part of the skin of the cheek itself [14, 15, 16].

**Figure 1:**
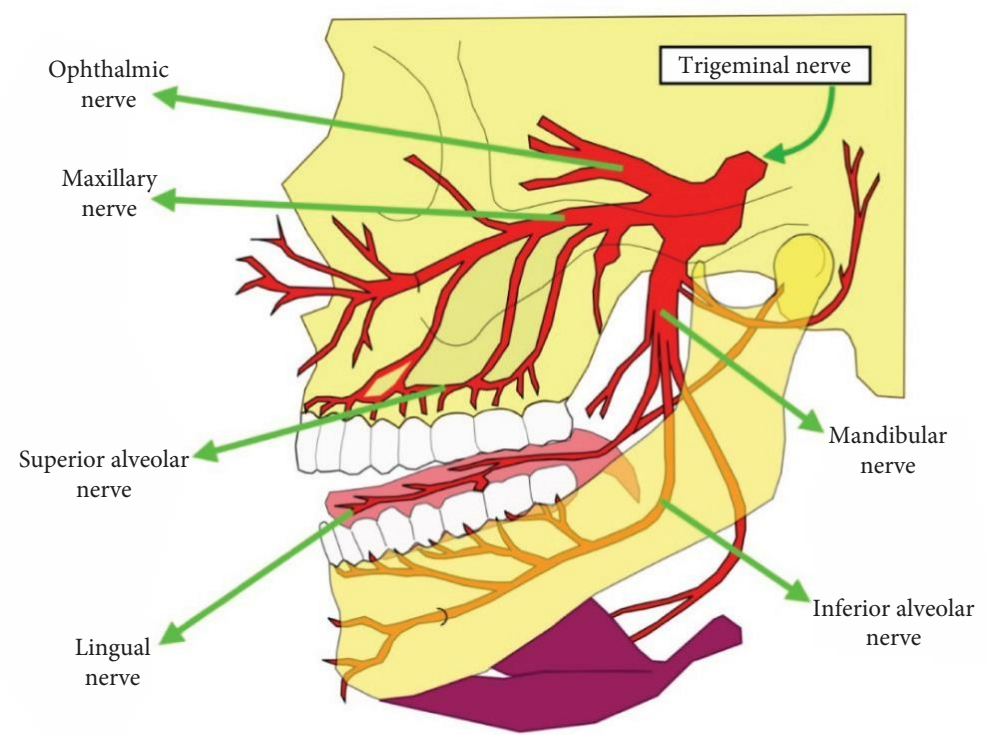
The ophthalmic nerve enters the superior orbital fissure, the maxillary nerve goes through the round hole, and the mandibular nerve escapes via the oval hole of the sphenoid bone.

It is important to note that the mandibular nerve, as part of the TN, is involved in the transmission of sensory signals from the facial region to the brain, contributing to the perception of sensitivity and response to various stimuli coming from these areas of the face (Figure 1) [17].

This is the anatomy of the TN, which has three branches that exit the skull at separate sites [18, 19].

**Figure 2:**
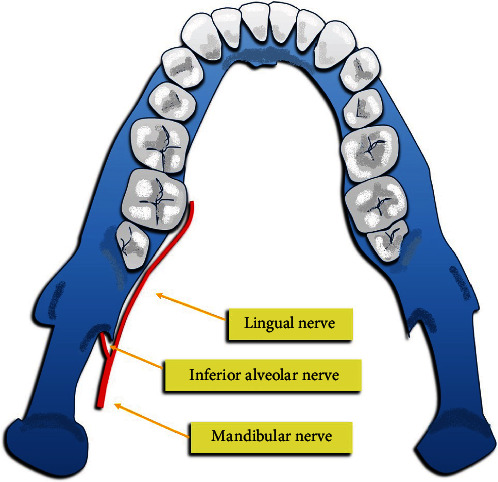
Course of the mandibular nerve with its branches, lingual and inferior alveolar nerve.

The IAN travels in an unusual way within the mandibular bone, but its structure can promote good recovery after damage, while the LN is more vulnerable, since if damaged, it has less chance of complete regeneration due to its more exposed position and structure (Figure 2) [20, 21].

**Figure 3:**
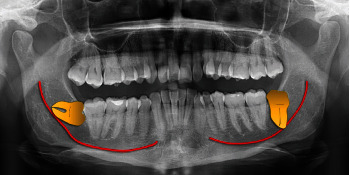
A radiographic example of the possible anatomical relationships between IAN and M3.

The anatomical relationship between the IAN and M3 can be assessed radiographically to predict the risk of damage (Figure 3). Some radiographic factors have been linked to IAN injury, but the presence of these factors does not always accurately predict actual damage [22, 23].

Injuries to the LN are varied, and their incidence varies widely. They may depend on the surgical technique, and raising and retracting a lingual mucoperiosteal flap have been associated with increased LN damage [24, 25, 26]. In general, the incidence of injury to the IAN, during dental procedures such as tooth extraction, varies between 0.2% and 5% [27, 28]. Regarding lesions in the LN, the incidence is generally reported to be lower than in the IAN, with estimates varying between 0.4% and 0.6% [28].

However, it is important to note that these figures can be influenced by numerous factors and may vary based on the specific nature of the surgery and individual patient characteristics [29, 30, 31]. Therefore, an accurate assessment of the risk of nerve injury during dental procedures requires a thorough case evaluation by the surgeon [32, 33, 34]. Other risk factors include the patient's age and tooth impaction [35, 36, 37, 38].

The buccal nerve, if damaged, can cause anesthesia of the cheek. Its structure and location close to the M3 make it vulnerable, especially during surgery involving the removal of the bone [39].

Complications associated with this nerve can emerge from several factors, including trauma, disease, or medical procedures. Some of these complications are [40, 41] as follows:

1. Mandibular nerve neuropathy: this condition involves damage or injury to the mandibular nerve, which can result from direct trauma, surgery to the jaw or surrounding structures, localized infections, or chronic compression of the nerve [42]. This can cause numbness, tingling, or pain in the area innervated by the nerve, affecting sensation in the jaw, cheek, and surrounding area [15, 43, 44].
2. Peripheral neuropathy: this can occur when the mandibular nerve suffers damage or injury near its peripheral branches. This can lead to conditions such as trigeminal neuralgia, in which the patient experiences intense, debilitating episodes of excruciating pain in the maxillary, mandibular, or facial region [41, 45]. Often, the pain is due to inflammation of the mandibular or maxillary nerve; more rarely, it is due to the ophthalmic nerve. In most cases, neuralgia is caused by compression of the nerve root by a blood vessel [46, 47]. Compression is responsible for the demyelination of nerve fibers. When myelin, a sheath that surrounds the nerve fiber, is missing, the nerve becomes hypersensitive to stimuli, and for this reason, neuralgia occurs [25, 48]. A noninvasive curative treatment is trigeminal neurolysis with targeted fluoroscopy, a minimally invasive therapy performed with a sedated patient. It is a safe, rapid, and often decisive treatment [49, 50].
3. Surgical complications: during surgery on the jaw, face, or surrounding structures, the mandibular nerve can be accidentally damaged [51, 52, 53]. This can cause postoperative symptoms such as persistent numbness or tingling in the affected area, with possible impairment of sensory and motor function [5, 54, 55]. Some dental conditions or dental procedures can also affect the mandibular nerve, such as the extraction of wisdom teeth in the lower jaw region which can pose a risk of injury to the mandibular nerve, leading to tingling, numbness, or pain in the surrounding area [56, 57, 58]. Implantology procedures [5] can lead also to nerve complications: insertion torque and sample size of implants should be adequately chosen [59, 60, 61].
4. Side effects from medical treatments, such as chemotherapy or radiation therapy, for tumors in the head/neck region. Radiation therapy, in particular, can cause damage to surrounding tissue, including the nerve, leading to neuropathy and associated symptoms [62, 63].
5. Synovitis of the temporomandibular joint (TMJ): inflammation of the TMJ can compress or irritate the mandibular nerve, causing pain, numbness, or dysfunction in the affected area [45, 64].

TN complications can range from mild to severe, affecting the patient's quality of life. Treatment depends on the underlying cause and may include drug therapies for pain, surgery to repair injury or damage, rehabilitation therapies, and symptom management to improve the patient's function and comfort [65, 66, 67].

It is important to avoid these injuries with adequate procedures [68] and properly manage patients with nerve injuries, including accurately diagnosing the type of injury, monitoring recovery, and treating appropriate cases [69, 70, 71].

Some authors have drawn up management protocols for lesions of the inferior alveolar and LN in the M3 extraction surgery.

For the IAN, they are as follows:

- Compression and section during surgery, if the IAN is compressed or cut, management depends on the severity of the injury [72].
- First degree (neurapraxia): mild compression of the nerve can cause a temporary block of nerve conduction. Usually, this leads to a complete recovery [73].
- Second degree (axonotmesis): compresses or crushes the nerve causing degeneration of the axons beyond the site of injury. Recovery depends on the regeneration of damaged axons [74].
- Third, fourth, and fifth degrees (more severe injuries): more serious injuries may require surgery to repair the nerve, especially if a complete section of the nerve occurs [35, 75].

For LN, they are as follows:

- LN rupture: if the LN is damaged during extraction, treatment depends on the severity of the injury [76].
- Minor injuries: in some cases, minor injuries may not require intervention, but monitoring is essential [10, 77].
- More serious injuries: if sensory loss persists or complete anesthesia occurs, radiographic investigation may be necessary to evaluate the damage and possible surgical repair [78, 79, 80].

The treatment protocols are as follows:

- Evaluation: postoperative evaluation is essential to monitor sensory recovery in patients [81].
- Surgical treatment: for severe or persistent injuries, especially if diagnosed within a certain postoperative period, restorative surgeries may be considered to facilitate sensory recovery [50].
- Repair surgery procedures: from neurolysis (freeing trapped nerves) to direct repair of the nerve with sutures [82, 83].

Monitoring patients, sensory testing, and observing recovery are key to determining whether surgery is needed and what type of surgery will be best if nerve damage occurs [84, 85]. Prevention of neurological complications begins with detailed preoperative planning. Advanced surgical techniques, such as the use of lasers and the piezoelectric approach, have been developed to reduce trauma to nerve tissue. In addition, rigorous postoperative follow-up is crucial for early diagnosis and timely management of complications [86].

In conclusion, while lower molar disinclusion surgery is a relatively common procedure, it requires careful consideration of potential neurological complications. The combination of accurate preoperative evaluation, modern surgical techniques, and effective postoperative management can help minimize risks and improve outcomes for patients [87].

The aim of this study is to investigate the neurological complications resulting from lower molar disinclusion surgery. In particular, we intend to evaluate the frequency, nature, and risk factors associated with lesions of the mandibular nerve and its branches during and after the surgical procedure [88]. This study also aims to compare the effectiveness of preoperative radiographic techniques, such as computed tomography (CT) and panoramic radiography, in preventing nerve damage. Furthermore, it aims to examine postoperative management strategies to minimize sensorineural dysfunction and improve patients' quality of life. Through a detailed analysis of injury mechanisms and predisposing factors, the study aims to provide clinical guidelines to reduce the risk of neurological complications in the future.

## 2. Materials and Methods

### 2.1. Protocol and Registration

The Preferred Reporting Items for Systematic Reviews and Meta-Analyses (PRISMA) protocols were followed when conducting this review, and the protocol was registered at PROSPERO under the ID: 496565.

### 2.2. Search Processing

We searched PubMed, Scopus, and Web of Science with a constraint on English language papers from January 1, 2003, to September 30, 2023, that matched our topic. The following Boolean keywords were utilized in the search strategy: mandibular nerve complications AND oral surgery (Table 1). These terms were chosen because they best described the goal of our inquiry, which was to learn more about postsurgical neurological complications in extractive surgery of the lower molars.

### 2.3. Eligibility Criteria and Study Selection

Research focusing on the impact of nerve complications postsurgery were chosen for analysis. The selection process involved two phases: firstly, evaluating titles and abstracts, followed by a thorough examination of full-text articles. Articles meeting the following criteria were included: (a) human-based studies, (b) comparative analysis of treatments with other interventions, and (c) availability of full-text content in English. Reviews, meta-analyses, and studies involving animals were excluded from consideration. The titles and abstracts from the initial search were collected and reviewed for relevance. Full publications of pertinent research were acquired for further assessment. Two different reviewers appraised the gathered studies based on the specified criteria to determine their inclusion.

### 2.4. Data Processing

Two independent reviewers conducted a database search to identify pertinent studies using predetermined selection criteria. The quality assessment of the chosen articles was also independently performed by these reviewers. Selected articles were stored in Zotero (version 6.0.15). Any disparities between the reviewers were resolved by consulting a senior reviewer (F.I.). The selection procedure and a summary of the chosen records are presented in Figure 3 and Table 2, respectively.

### 2.5. Quality Assessment

The quality of the included papers was assessed by two reviewers, E.d.R. and A.D.N., using the ROBINS which is a tool developed to assess risk of bias in the results of nonrandomized studies that compare health effects of two or more interventions. Seven points were evaluated, and each was assigned a degree of bias. A third reviewer (F.I.) was consulted in the event of a disagreement until an agreement was reached. The question in the domains evaluated in the ROBINS is the following:

  Bias due to confounding
  Bias arising from measurement of exposure
  Bias in the selection of participants into the study
  Bias due to postexposure intervention
  Bias due to missing data
  Bias arising from measurement of the outcome
  Bias in the selection of the reported results.

### 2.6. PICOS Criteria

Table 2 illustrates the components of the PICOS framework (Population, Intervention, Comparison, Outcome, Study Design), outlining the criteria for population, intervention, comparison, outcomes, and research design utilized in this assessment.

## 3. Results

**Figure 4:**
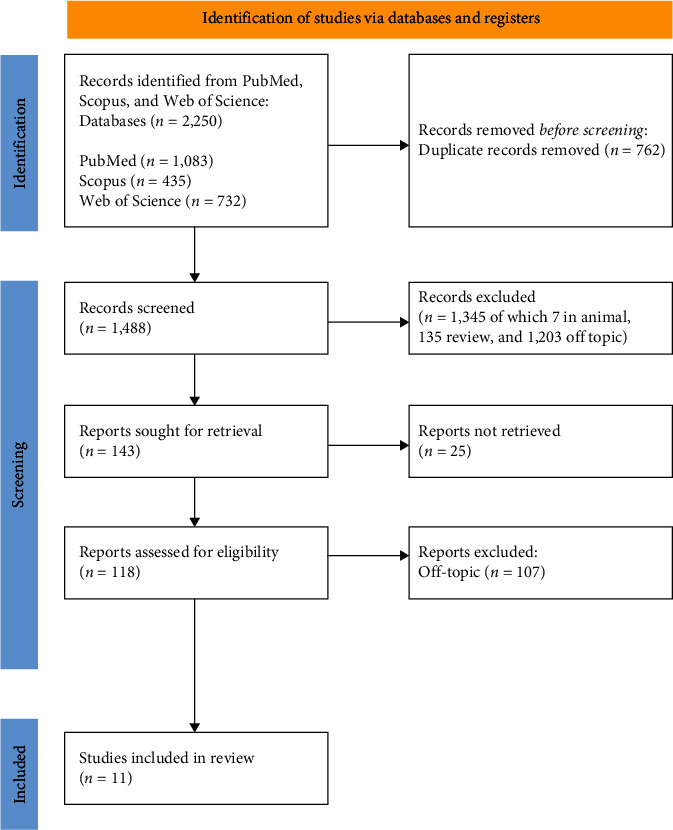
PRISMA flowchart.

There were 2,250 entries altogether from the first search (1,083 from PubMed, 435 from Scopus, and 732 from WOS), and 1,488 articles remained after 762 duplicates were eliminated. However, 1,345 of them were eliminated because of 135 reviews and meta-analysis, seven animal-related articles, and 1,203 off-topic articles. The other 25 reports were eliminated because they were not retrieved. Eleven records were deemed eligible after an additional 107 publications were eliminated from the list due to insufficient interest in the presented data (Figure 4). Each study's findings were presented in Table 3.

### 3.1. Quality Assessment and Risk of Bias of Included Articles

**Figure 5:**
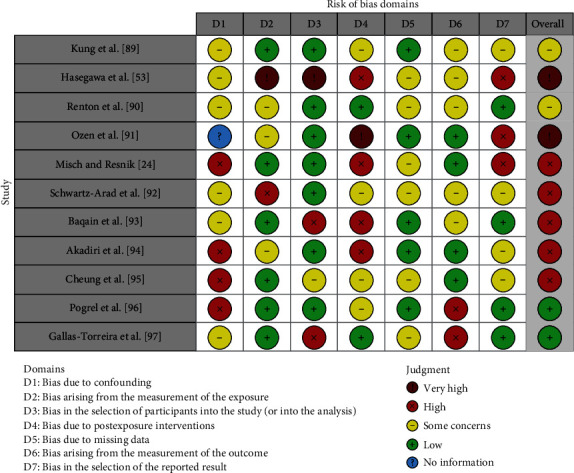
Bias assessment evaluated by ROBINS.

The risk of bias in the included studies is reported in Figure 5. Regarding the bias due to confounding, most studies have a high risk. The bias arising from measurement is a parameter with low risk of bias. Many studies have a low risk of bias due to bias in the selection of participants. Bias due to postexposure cannot be calculated due to high heterogeneity. The bias due to missing data is low in many studies. Bias arising from measurement of the outcome is low. Bias in the selection of the reported results is high in most studies. The final results show that five studies have high risk of bias, two have a very high risk of bias, and three have low risk of bias.

## 4. Discussion

**Figure 6:**
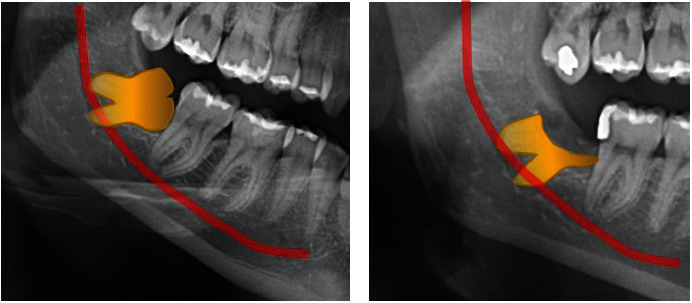
Clinical case in which a M3 coronectomy was performed, due to the very close relationships between M3 roots and IAN: (a) radiographic image before surgery and (b) postsurgery radiographic image.

Knowing the anatomy and histology of nerves provides a more detailed understanding of the correlation between the structure of the nerves themselves and the possible impacts of injury on nerve tissue. One of the cranial nerves most subject to trauma is the mandibular nerve, a branch of the TN, the largest of the cranial nerves [98, 99]. It is responsible for carrying sensory sensations from the lower part of the lip, chin, lower teeth, and associated soft tissues, as well as from the bone tissue of the jaw and some areas of the external ear. Anatomically, the mandibular nerve is composed of connective tissue and neural components [100]. The smallest functional units in a TN are the nerve fibers, which can be myelinated or unmyelinated. The more abundant myelinated nerve fibers consist of a single axis enclosed by a Schwann cell. These fibers and Schwann cells are surrounded by a protective layer of endoneurial connective tissue, composed of basal lamina, collagen fibers, and endoneurial capillaries. These nerve fibers are grouped into bundles, surrounded by a thin, dense layer of connective tissue called the perineurium, which maintains intrafascicular pressure and acts as a diffusion barrier to protect the individual fibers. Injury to these extraneural tissues can impair nerve transmission of individual fibers, leading to sensory disturbances. The extent of damage to each tissue type can determine the degree of sensorineural impairment. A primary concern related to injuries to the IAN is alteration of sensory functions such as touch, pressure, temperature, or pain. Loss of sensation in the cheek or lower lip can cause soft tissue damage when chewing and affect the ability to drink. Furthermore, the lack of sensation can cause significant pain, becoming debilitating for the patient [24]. One of the most frequent side effects of surgical removal of M3 is IAN injury which supplied sensation to the oral and facial regions. IAN injuries occur with an incidence ranging from 0.26% to 8.4% of cases, with most of these injuries being temporary and resolving within 6 months. In comparison, lesions to the LN have an incidence ranging from 0.1% to 22% of cases, with a good percentage of these that are also temporary, but with more cases that can become permanent than IAN. Most injuries to both nerves resolve within a few weeks or months, but a small number of patients may experience permanent sensory deficit [91]. Preoperative radiographical examination can prevent the occurrence of injury to the peripheral branches of the TN during surgical procedure [101]. Hasegawa et al. compared CT with panoramic radiographs on 2,528 teeth extracted of patients with a mean age of 36.2 ± 12.2 years using univariate and multivariate analyses. According to this comparative study, CT is able to prevent risk of nervous damage more accurately than orthopantomography through the presence of radiographic findings such as darkening of the root where it crosses the inferior alveolar canal, interruption or obliteration of cortical lines which delimit the inferior alveolar canal, and diversion or bending of the inferior alveolar canal in contiguity with the root apices [53, 102]. Schwartz-Arad et al. [92] focused, in 2018, on the complications that occur after the extraction of M3 in the jaw. During a 10-year period (2001–2011), 1,038 extractions were performed on 500 patients, mainly mandibular molars. Approximately 16.9% of cases presented postoperative complications, mainly in the form of dry abscesses (11.6%) and other symptoms such as inflammation, pain, hematoma, and temporary loss of sensation in some areas of the face. The severity of the complications is related to the degree of impaction of the tooth: completely or partially impacted molars show a higher incidence of complications than completely erupted ones. Additionally, smokers have a higher risk of complications, especially dry abscesses, than nonsmokers. Age, gender, side of extraction, and use of contraceptives do not appear to be factors significantly related to postoperative complications [95]. In conclusion, it is highlighted that smoking, the degree of impaction of the tooth, and the age of the patient can influence complications after the extraction of mandibular M3. It also recommends the use of specific antibiotics to reduce the risk of dry abscesses, especially in extractions of partially erupted teeth [92]. Baqain's [93] study conducted in 2008 also focuses on the frequency and risk factors of postoperative complications after surgical extraction of mandibular M3. The work examined several predictive variables and associated them with the complications encountered, including alveolar osteitis, trismus, and postoperative pain. Key findings and conclusions include alveolar osteitis with higher incidence in older individuals, when the side of the M3 differs from the operator's dexterity and with lingual retraction during surgery. Lingual retraction has been identified as an independent risk factor, associated with greater trauma of the operation. The greater risk in deeper impacts, especially totally bony ones, is trismus. Longer operative time was associated with a greater risk of prolonged pain. Advanced age has been linked to increased postoperative complications, especially alveolar osteitis. The correlation between the side of the M3 and operator dexterity suggests that removal of the M3 on the operator's nondominant side may be more complex. As final thoughts, alveolar osteitis has been identified as a common complication impacting productivity. The use of multivariable regression models allowed us to better identify risk factors. In summary, identifying these risk factors can guide the management of postoperative complications in the removal of mandibular M3, offering useful information for patients and healthcare professionals [93]. Other authors, such as Akadiri et al. [94], in 2009, they discussed sensorineural complications related to the surgical extraction of mandibular M3. Seventy-nine patients were evaluated for IAN, LN, and buccal (BN) nerve injuries through pre- and postoperative testing to determine incidence and duration. We sought to identify radiographic and operative risk factors associated with these injuries. The reported incidence of lesions was 6.6% for IAN, 2.6% for LN, and 4.0% for LBN, with most lesions resolving within 2 weeks. Depth of impaction and linguoversion emerged as significant risk factors for IAN and LN injuries, respectively, while no risk factors were identified for BN injuries. Most lesions were transient and resolved within a few weeks. The study also highlights the limitations of 2D imaging techniques for evaluating impacted M3. Some significant intra- and postoperative events were related to the observed nerve lesions. For example, patients with prolonged intra-alveolar bleeding have experienced injury to the IAN. Injuries to the BN have been traced to possible trauma during the surgical procedure [94]. For the prospective study by Cheung et al. [95] in 2010, conducted on extractions of the lower M3, it involved 3,595 patients subjected to 4,338 extractions, detecting a development of 0.35% of deficit in the IAN and 0.69% in the LN. Distoangular impaction emerged as a significant risk factor for LN deficit, while depth of impaction was associated with the risk of IAN deficit. Operator experience influenced the results, with dental students accounting for more LN deficits. Factors such as gender, age, lingual flap elevation, protection of the LN with a retractor, removal of the distolingual cortex, sectioning of the tooth, and difficulty in elevating the tooth were not significantly related to IAN or LN lesions [95]. Recovery was more significant at 3 months postsurgery for the IAN deficit and at 6 months for the LN deficit. At the end of the follow-up period, 66.7% of IAN deficits and 72.0% of LN deficits fully recovered. The study highlighted the importance of distoangular impaction and operator experience as significant risk factors for postoperative neurosensory deficits. Overall incidences of deficits were low, 0.35% for IAN and 0.69% for LN, with no significant differences related to age or sex. Furthermore, the recovery of deficits was monitored over time, finding that the majority of patients achieved complete recovery within the first 6 months postsurgery. The percentages of permanent sensorineural deficits were estimated to be 0.12% for the IAN and 0.16% for the LN, low values compared to other studies [95]. A study was conducted to avoid or reduce the risk of complications resulting from lower M3 extraction caused by injury to the lower alveolar nerve, which occurs in up to 3.6% of cases. The following were selected for this randomized clinical trial: 128 patients who required extraction of the mandibular M3 and who had radiological evidence of the proximity of the M3 to the IAN canal. Of these, 102 underwent extraction, while 94 underwent failed or successful coronectomy. During the planned coronectomy, some roots became loose and were removed, creating two groups of failed (36) or successful (58) coronectomy. The follow-up period lasted only about 2 years. Nineteen patients' nerves sustained injury following extraction, none following a successful coronectomy and three following a successful coronectomy. The study conducted therefore examines the effectiveness of coronectomy in maintaining, and therefore reducing damage, the IAN (Figure 6) [90].

The text by Gallas-Torreira et al. [97] of 2003 discusses paresthesias of the inferior dental nerve, which can result from various factors such as trauma, tumors, connective tissue diseases, infections, or idiopathic lesions. The most common cause is traumatic neuropathy of the IAN, often linked to extraction of the lower M3. Nerve injury even after endodontic treatments is a rare but possible complication as highlighted in a case of a 45-year-old woman who complained of pain and numbness after endodontic treatment on a lower left molar. After discovering that a gutta-percha tip had penetrated the dental nerve, surgical removal of it was performed. Although the pain disappeared after removal, the numbness persisted but then gradually resolved. Direct or indirect mechanical trauma, nerve compression due to hematoma or root canal filling materials, and chemical damage due to intraductal toxic substances are some of the mechanisms of nerve injury during endodontic treatment. Finally, we emphasize the importance of timely elimination of the cause of the nerve injury and control of inflammation to manage paresthesia [24]. The risk of damage to the nerves of the face can occur not only during extraction surgery of the lower molars, but also during other surgical procedures [35]. Misch and Resnik [24], in 2012, for example, examined potential complications related to implant surgery involving the sensory nerves connected to the TN. The most commonly affected nerves during implant surgery are the IAN and its mental branch, as well as others such as the LN, buccal nerve, and infraorbital nerve, due to their anatomical location. Nerve injuries can occur during different phases of surgery, from anesthesia administration to implant placement and soft tissue swelling after surgery. The incidence of these lesions can vary greatly, from 0 up to 44%. When nerve damage occurs, it is important for the provider to recognize the type and extent of the damage to provide the best postoperative care. Traumatic or iatrogenic complications may involve total or partial nerve resection, pinching, stretching, or entrapment of the nerves, resulting in sensory deficits ranging from mild loss of sensation to permanent painful dysfunction [24]. The paper conducted by Pogrel et al. [103] in 2011 examined patients who suffered permanent damage to the inferior alveolar or LN due to dental treatments. The study interviewed patients 3–9 years after the accident from the Oral and Maxillofacial Surgery Clinic at the University of California, San Francisco. Of the 727 eligible patients, 145 completed surveys [103]. Many have sought both conventional and alternative treatments, and some have undergone surgery elsewhere. The results showed significant impacts on the daily lives of patients, including employment, relationship problems, depression, speaking, and eating difficulties. However, over time, most patients experienced improvement, often adopting various coping mechanisms. Men tended to report greater reduction in symptoms than women, while patients over 40 reported more long-term pain than younger ones. The LN showed greater improvement than the alveolar nerve, although complete recovery was rare. Although long-term problems persisted, most patients showed modest improvement over time, often attributable to adaptation rather than actual medical improvements [96]. Further research could explore less common complications, such as infections and sensorineural deficits [93]. The text of Ozen et al. [91], in 2006, discusses a study that focuses on the use of low-level laser (LLL) therapy in patients who have experienced nerve injuries following the extraction of mandibular M3. These injuries are commonly associated with the roots of impacted mandibular molars and the IAN. While these issues occur in a frequency range of 0.4%–8.4%, most cases are temporary, but some can result in long-term and even permanent incapacity if they persist beyond 6 months. The study presents the results of employing low-level laser therapy (LLLT) to address these nerve injuries. Specifically, four female patients with paresthesia and dysesthesia in various areas (lip, chin, gingiva, and buccal areas) underwent clinical neurosensory tests before and after the therapy. These tests included assessments like brush stroke directional discrimination, two-point discrimination, and subjective evaluations using a virtual analog scale. The results were then tracked over time. The study observed a significant improvement in the speed and extent of neurosensory recovery when comparing the scores from neurosensory assessments after LLL therapy with the initial values before treatment. This suggests that low-intensity laser therapy could expedite the return of neurosensory function following M3 surgery. In conclusion, the study indicates that LLLT shows promise in reducing chronic sensory nerve impairment resulting from M3 surgery. However, it also highlights the need for further research to better understand and optimize the therapeutic potential of this treatment option [91]. The text by Gallas-Torreira et al. [97] of 2003 discusses paresthesias of the inferior dental nerve, which can result from various factors such as trauma, tumors, connective tissue diseases, infections, or idiopathic lesions. The most common cause is traumatic neuropathy of the IAN, often linked to extraction of the lower M3 [104]. Nerve injury even after endodontic treatments is a rare but possible complication as highlighted in a case of a 45-year-old woman who complained of pain and numbness after endodontic treatment on a lower left molar. After discovering that a guttapercha tip had penetrated the dental nerve, surgical removal of it was performed. Although the pain disappeared after removal, the numbness persisted but then gradually resolved. Direct or indirect mechanical trauma, nerve compression due to hematoma or root canal filling materials, and chemical damage due to intraductal toxic substances are some of the mechanisms of nerve injury during endodontic treatment [105]. Finally, we emphasize the importance of timely elimination of the cause of the nerve injury and control of inflammation to manage paresthesia [97]. Injuries to the IAN during implant surgery usually occur after the nerve enters the lingula of the mandibular ramus and along its path into the body and/or as it exits the mental foramen. Injuries to these tissues can affect the transmission of sensations and lead to neurosensory disorders: changes in touch, pressure, temperature, and pain. The terminology used to describe sensory alterations is paresthesia, dysesthesia, and anesthesia, with the aim of standardizing the description of neurosensory deficits:

1. Paresthesia is a sensory alteration that is not necessarily painful. Typically, paresthesia involves abnormal sensations such as tingling, numbness, or a “pins and needles” sensation. Although it is a change in sensitivity, it does not necessarily cause discomfort or pain [106].
2. Dysesthesia includes abnormal or unpleasant sensory sensations, often described as painful, uncomfortable, or unpleasant. It may include burning, stinging, aching, or numbness sensations that may be constant or intermittent [107].
3. Anesthesia means complete loss of sensitivity or sensation. In this case, the affected area completely loses the ability to perceive touch, pressure, temperature, or pain. Anesthesia results in a total lack of sensation [108].

Many factors, including the type of injury and the patient's age and gender, influence the neurological response to an injury [109]. Pharmacological therapy with corticosteroids and nonsteroidal anti-inflammatories is recommended to reduce inflammation and minimize neurological complications [110]. For other possible pharmacological treatments, such as antidepressants, anticonvulsants, and topical anti-inflammatory agents, we emphasize the need for caution in the use of such drugs and management by clinicians experienced in nerve injury. Carrying out a timely and accurate assessment of these injuries and sending them to specialists in nerve injuries based on the severity and type of injury allows the resolution, in most cases, of postsurgical nerve pathologies [24]. In order to reduce complications during surgical procedures with symphysis block, the retrospective study by Kung et al. [89], in 2017, evaluated the location, incidence, and dimensions of the mandibular lingual canal and anterior loop using CT cone beam. Three-dimensional images are of enormous help in planning surgical interventions, particularly in the mandibular area. This allows for safer and more predictable procedures. Three-dimensional images of 215 patients (105 men and 110 women) were used to examine the median lingual canal, symphysis bone thickness, and anterior loop length. Each patient had at least one median lingual canal in the symphysis, and the diameter of the major ramus ranged between 0.21 and 1.48 mm (mean 0.85). The study brought to light statistically significant gender differences in the lingual canal: longer in males than in females. The finding implies that, due to the numerous and complex anatomical differences, routine CT scans are required before any surgical treatment in the symphysis region [89].

### 4.1. Limitations of the Study

Many data come from limited and specific samples, reducing the generalizability of the findings. Short-term follow-up in some studies does not allow full evaluation of long-term complications. The use of various methods to evaluate complications may introduce inconsistencies in the results. Some studies do not adequately consider all confounding factors, such as pre-existing medical conditions or operator experience. Two-dimensional images may be insufficient compared to three-dimensional ones for precise risk assessment. There is a risk of bias in observational studies, affecting the reliability of the results [111]. The low diversity in the samples can limit the representativeness of the results. Less common complications may be underreported due to the rarity of events. The prevalence of observational and retrospective studies limits the ability to reliably determine causality. These limitations highlight the need for further research with larger samples, longer follow-ups, and more rigorous study designs to improve the understanding and management of postoperative neurological complications [112].

## 5. Conclusions

Complications related to the mandibular nerve, the largest branch of the fifth (V) cranial nerve, can arise from various sources such as surgical procedures, diseases, or trauma. Procedures like impacted third molar (M3) extraction, endodontic treatments, implant placements, postinfection or trauma neuropathy, synovitis of the temporomandibular joint, or medical interventions may lead to involvement or damage of the mandibular nerve, encompassing the IAN and lingual branches. In assessing the risk and progression of nerve damage, CT scans might offer more precise predictions compared to panoramic imaging. A comprehensive preoperative evaluation of potential risks associated with surgical procedures aids in planning microsurgical interventions to minimize neurosensory damage risks to the mandibular nerve. Nonetheless, patients should be fully informed preoperatively about potential postoperative complications as part of the informed consent process. Should hypesthesia or dysesthesia of the mandibular nerve occur as a surgical complication, continuous monitoring via neurosensory tests is essential, with possible referral to neurosurgery. Various treatments exist for nerve damage, including LLLT, pain management medications, or physical therapy. While these therapies may improve neurosensory impairment, patients often report a decline in their quality of life. Surgeons should prioritize preventing such occurrences through thorough preoperative investigations. Conservative therapies can be attempted to ameliorate this medical condition. This manuscript emphasizes the importance of detailed understanding of nerve anatomy to prevent and manage postsurgical neurological complications associated with lower molar disinclusion. It provides an overview of frequent mandibular and LN injuries during lower third molar extraction and proposes preventive and therapeutic measures based on recent clinical studies.

This research highlights specific risk factors for postsurgical neurological complications. The use of LLLT for the treatment of nerve injuries helps to improve clinical practice and reduce postoperative complications.

Dental professionals can apply the knowledge gained from this manuscript in their clinical practice through more accurate surgical planning and the adoption of advanced surgical techniques to minimize the risk of nerve injury. In addition, the implementation of more detailed radiographic examinations, such as CT, can improve the prediction and prevention of complications. Awareness of potential complications and their management enables clinicians to adequately inform patients and provide targeted postoperative follow-up.

## Figures and Tables

**Table 1:** Indicators for database searches.

Article screening strategy
Keywords: A, mandibular nerve complications; B, oral surgery
Boolean indicators: A AND B
Timespan: 2003–2023
Electronic databes: PubMed; Scopus; WOS

**Table 2:** PICOS criteria.

Criteria	Application in the present study
Population	Adults
Intervention	Extractive surgery of the lower molar
Comparisons	Total extraction of the third molar compared with coronectomy of the third molars, analyzing the risk factors for IAN injury
Outcomes	Recovery periods of neuronal lesions caused by surgical procedures
Study design	Clinical trials and retrospective studies

**Table 3:** A descriptive item selection summary.

Authors (year)	Type of the study	Aim of the study	Materials	Results
Kung et al., 2017 [89]	Retrospective study	Using the modeling and visual interpretation of cone-beam CT, this retrospective study assessed the location, incidence, and dimensions of the mandibular lingual canal and the anterior loop in the Taiwanese population.	There were 215 patients in the sample group (105 men and 110 women, with a mean age of 57 years). Cone-beam CT and three-dimensional reconstructed images were used to identify and determine the anterior loop length, median lingual canal, and symphysis bone thickness. Unpaired statistical analysis was used to evaluate and examine the correlation of all the data for both men and women.	Every patient had at least one median lingual canal in the symphysis, and the major branchʼs diameter varied between 0.21 and 1.48 mm (mean, 0.85 mm), with statistically significant gender differences (longer in males than in women). Neurovascular damage was shown to be 13.0% likely at a harvesting depth of 4 mm for the distance from the buccal bone to the terminal end of the median lingual canal; this risk was significantly higher in women (19.1%) than in males (6.7%).

Hasegawa et al., 2013 [53]	Comparative study	Compare panoramic radiographs and computed tomography (CT) in investigating the relationships among the risk factors for IAN injury.	Univariate and multivariate analysis on 2,528 surgical avulsion of mandibular M3 of patients with a mean age of 36.2 ± 12.2 years performed by dentists at the Department of Oral and Maxillofacial Surgery.	CT findings may be able to predict the development of IAN injury more accurately than panoramic findings.

Renton et al., 2004 [90]	Randomized controlled clinical trial	Randomization of 128 patient who needed M3 extractions and with radiological evidence of the M3 closeness to the IAN canal.	The patients (128) were subjected to two different operations, extraction (102) and coronectomy (94), and coronectectomy in two soubgroups, successful coronectomy (*n* = 58) and failed coronectomy (*n* = 36). Follow up lasted an average of 25 (13) months.	Following extraction, 19 nerves (19%) were injured, none following a successful coronectomy and three after failed coronectomy. This is the first clinical trial of the efficacy of coronectomy in preserving the IAN.

Ozen et al., 2006 [91]	Clinical study	Report of the effects of LLT in four patients with lomgstanding sensory after M3 extraction.	LLT was applied to four female patients who underwent M3 surgery and had chronic nerve damage.	There was a notable acceleration of the time course and degree of neurosensory return when the neurosensory assessment scores following LLL therapy treatment were compared with the baseline values before to treatment.

Misch and Resnik, 2012 [24]	Clinical study	Establish a policy designed especially for general dentists to handle cases when a nerve has been damaged during bone grafting or implant surgery.	The procedure outlined in this article is broken down into five phases: (1) nerve damage is suspected during surgery; (2) nerve transection is known; (3) 1 week follows the procedure; and (4) 12 weeks.	It is recommended to follow the right course of therapy (pharmacology, monitoring, etc.) during each period, and when necessary, to refer the patient to a nerve expert.

Schwartz-Arad et al., 2018 [92]	Retrospective study	Examine the relationship between risk variables and the incidence of problems after M3 extraction.	463 individuals who had their mandibular M3 extracted between 2001 and 2011 (by one surgeon, DSA) were included in the research. 665 mandibular M3 were removed in total. The patients varied in age from 13 to 75 years old, with a median age of 26 years and an average age of 29 ± 11.30 years. Medical and general data were gathered from patient records.	Age, the degree of impaction, the side of the extraction, and cigarette smoking all raise the risk of complications following mandibular M3 extraction.

Baqain et al., 2008 [93]	Prospective cohort study	This study sought to determine the risk factors and approximate the incidence of postoperative problems following surgery on the mandibular M3.	Three groups of predictive factors were identified: anatomical, surgical, and patient-specific. Postoperative complications, recorded as present or absent, were considered as outcome variables. After bivariate analysis, a multivariable logistic regression model was used to identify independent predictors of the most frequent postoperative problems.	Longer operations, deeper impaction, M3 side dissimilarity from the operator's handedness, and advanced age all contribute to increased postoperative morbidity.

Akadiri et al., 2009 [94]	Clinical study	The study aimed to record the incidence and duration of inferior alveolar, lingual, and buccal nerve damage after extraction of wisdom molars, identifying associated radiographic and operative risk factors.	79 patients undergoing surgical extraction of unilateral lower judgment molars received sensorineural testing before and after surgery to evaluate the incidence and duration of complicated nerve lesions. Risk factors for nerve injury were identified among radiographic variables and documented operative events.	It is possible to predict nerve damage during M3 surgery based on some radiographic risk markers and some unanticipated intraoperative occurrences. The majority of injuries are just temporary in nature.

Cheung et al., 2010 [95]	Prospective study	To study the frequency of future neurosensory impairment resulting from damage to the LN and IAN, investigate potential risk factors, and characterize the healing process.	3,595 patients of different ages and genders underwent extraction of lower wisdom molars. Of a total of 4,338 extractions, 0.35% developed IAN deficiency and 0.69% LN deficiency. It was noted that distoangular impaction significantly increased the risk of LN deficiency, while the depth of impaction was related to the risk of IAN deficiency. College students more frequently caused LN deficits.	67% of IAN deficits and 72% of LN deficits were fully healed at the end of the follow-up period.

Pogrel et al., 2011 [96]	Clinical study	To analyze effects of permanent involvement of the inferior or lingual alveolar nerve due to dental treatments.	The study involved patients reviewed between 3 and 9 years after injury. Of 727 patients eligible for the study, 145 (95 women and 50 men) completed the telephone survey. Overall, patients experienced improvement over time, often using different coping mechanisms. Men showed a greater decrease in symptoms than women, while patients over 40 reported more long-term pain than younger patients.	Symptoms of the LN improved more than those of the IAN, but for most treated patients, there was improvement in symptoms over time.

Gallas-Torreira et al., 2003 [97]	Clinical study	To implement a therapeutic protocol for an endodontic complication that led to paresthesia of the IAN.	45-year-old woman who has had pain in the left half of her jaw and numbness in her left lip for 15 days. After endodontic treatment on a lower left molar, a portion of filling material was found beyond the apex of the tooth root, requiring tooth extraction. Despite the extraction, the pain and numbness did not decrease. The pain disappeared 15 days after the gutta-percha residue was removed, but the lip numbness persisted for about a month before resolving.	There are three potential pathways for nerve injury: heat, chemical, and mechanical. Compression, stretching, partial or complete resection, and laceration are examples of mechanical injuries. Axonotmesis, or discontinuity of the nerve due to Wallerian degeneration of the covering's distal and integrated fibers, or neurotmesis, or complete sectioning of the nerve, are the two possible outcomes of the injury.

## Abbreviations

CT: Computed tomography
IAN: Inferior alveolar nerve
IVRO: Intraoral verticosagittal ramus osteotomy
LN: Lingual nerve
LLL: Low level laser
M3: Mandibular third molar
TMJ: Temporomandibular joint
TN: Trigeminal nerve.
